# Chitosan Nanoparticles: An Alternative for In Vitro Multiplication of Sugarcane (*Saccharum* spp.) in Semi-Automated Bioreactors

**DOI:** 10.3390/plants14111697

**Published:** 2025-06-01

**Authors:** Eucario Mancilla-Álvarez, María Karen Serrano-Fuentes, María Angélica Fuentes-Torres, Ricardo Sánchez-Páez, Jericó Jabín Bello-Bello

**Affiliations:** 1 Postgraduate College-Campus Cordoba, Amatlan de los Reyes, Veracruz 94953, Mexico; euca_man90@hotmail.com (E.M.-Á.); ame_karen15@hotmail.com (M.K.S.-F.); 0105ftma@gmail.com (M.A.F.-T.); risa@colpos.mx (R.S.-P.); 2 SECIHTI-Postgraduate College-Campus Cordoba, Amatlan de los Reyes, Veracruz 94953, Mexico

**Keywords:** hormesis, photosynthetic pigment, phenolic, peroxidation, hydrogen peroxide, antioxidant capacity

## Abstract

Chitosan nanoparticles (CsNPs) are biocompatible, biodegradable, and non-toxic natural polymers at low concentrations with diverse applications in in vitro plant tissue culture. This study aims to evaluate the effect of CsNPs during in vitro multiplication of sugarcane (*Saccharum* spp.) using temporary immersion bioreactors. CsNPs were evaluated at concentrations of 0, 25, 50, 100, and 200 mg L^−1^ in Murashige and Skoog liquid culture medium. After four weeks of culture, response percentage, the number of shoots per explant, shoot length, number of leaves per explant, dry matter, chlorophyll content, *β*-carotene content, lipid peroxidation, phenolic content, hydrogen peroxide content, and antioxidant capacity were evaluated. The results showed that the highest response percentages were obtained in the treatments with 0, 25, and 50 mg L^−1^ CsNPs, whereas the lowest response percentages were obtained in the treatments with 100 and 200 mg L^−1^ CsNPs. Concentrations of 25 and 50 mg L^−1^ CsNPs promoted cell growth and differentiation, whereas 100 and 200 mg L^−1^ CsNPs inhibited it. Chlorophyll content increased by 25 and 50 mg L-1 CsNPs, whereas *β*-carotene content increased by 100 and 200 mg L^−1^ CsNPs. Lipid peroxidation and antioxidant capacity increased with increasing CsNP concentrations. The phenolic content increased by 100 mg L^−1^ CsNPs, whereas the hydrogen peroxide content decreased with increasing CsNP concentrations. In conclusion, CsNPs are an alternative for stimulating tissue growth and differentiation during the in vitro multiplication of sugarcane.

## 1. Introduction

Nanomaterials (NMs) are known for having at least one dimension in the nanometer scale (1–100 nm) [1]. NMs have applications in medical sciences, the environment, the food industry, and agriculture [2,3]. In agriculture, NMs have facilitated the development of new products such as nanopesticides, nanofertilizers, and growth nanoregulators aimed at improving agricultural production [3]. Nanomaterials used in agriculture include carbon-based (such as fullerenes and carbon nanotubes), metal-based (such as silver nanoparticles and gold nanoparticles), and metal oxide (such as titanium dioxide nanoparticles and zinc oxide nanoparticles) types [4,5]. Due to their nanometer scale, as well as their optical, electrochemical, and mechanical properties, nanoparticles (NPs) have different effects and applications for plant biotechnology [6,7]. Chitosan nanoparticles (CsNPs) have demonstrated multiple benefits in agriculture. They have been used to reduce salinity stress [8], mitigate heavy metal toxicity [9,10], control pests [11], act as microbicidal agents [12], and stimulate plant growth at low concentrations while causing inhibition or death at high or lethal concentrations (also known as the hormetic effect) [13]. An alternative way to study the effects of CsNPs on plants is through plant tissue culture. This technique allows the manipulation of cells, tissues, organs, or whole plants under aseptic and controlled conditions.

Studies have reported the application of CsNPs in in vitro culture of corn (*Zea mays*) [14], rice (*Oryza sativa*) [15], potato (*Solanum tuberosum*) [16], and fig tree (*Ficus carica*) [17]. The application of CsNPs improves pest and disease tolerance, modulates antioxidant capacity, increases phenolic content, reduces reactive oxygen species (ROS) accumulation [18], mitigates abiotic stress, promotes seed germination, and enhances growth [19] and cell differentiation [20]. However, the physiological and biochemical mechanisms by which CsNPs act on plants have not been fully elucidated. Therefore, this study aims to evaluate the effect of chitosan nanoparticles during in vitro multiplication of sugarcane (*Saccharum* spp.) cv. Mex 69–290 using temporary immersion bioreactors.

## 2. Results

### 2.1. Effect of CsNPs on In Vitro Multiplication

**Figure 1:**
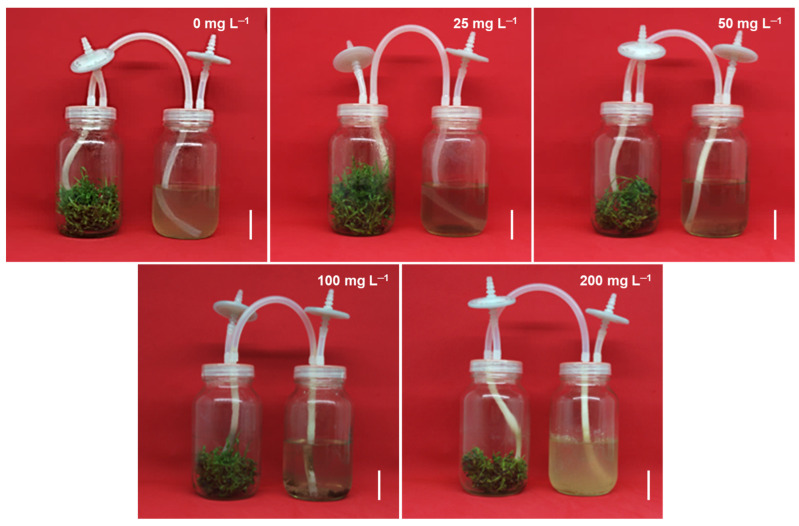
Effect of different chitosan nanoparticle concentrations on in vitro multiplication of sugarcane (*Saccharum* spp.) cv. Mex 69–290, after four weeks of culture in temporary immersion bioreactors (TIBs), left glass vessels with explants and right glass vessels with culture liquid medium. White bar = 5 cm.

The different CsNP concentrations had an effect on the response percentage and in vitro multiplication of sugarcane (Table 1). The highest response percentages were obtained in the treatments with 0, 25, and 50 mg L^−1^ CsNPs, obtaining 100% response in all explants, whereas the lowest response percentages were obtained in the treatments with 100 and 200 mg L^−1^ CsNPs, with 83 and 66% response, respectively. The highest number of shoots per explant was observed in the treatments with 25 and 50 mg L^−1^ CsNPs, whereas the lowest number of shoots was observed in 200 mg L^−1^ CsNPs. For the shoot length variable, the longest shoots were observed in the treatment with 25 mg L^−1^ CsNPs, whereas the shortest shoots were observed in the 100 and 200 mg L^−1^ CsNP treatments. Regarding the number of leaves per shoot, the shoots with the greatest number of leaves were observed in the 25 mg L^−1^ CsNP treatment, whereas the shoots with the fewest number of leaves were observed in the 200 mg L^−1^ CsNP treatment (Figure 1). Regarding the biomass variables, the highest fresh weight was observed in the 25 mg L^−1^ CsNP treatment, whereas the lowest fresh weight was observed in the 0, 100, and 200 mg L^−1^ CsNP treatments. The highest dry weight was observed in the 25 mg L^−1^ CsNP treatment, whereas the lowest dry weight content was observed in the 200 mg L^−1^ CsNP treatment. No significant differences were observed between treatments for the dry matter variable.

### 2.2. Effect of Csnps on Photosynthetic Pigment Contents

**Figure 2:**
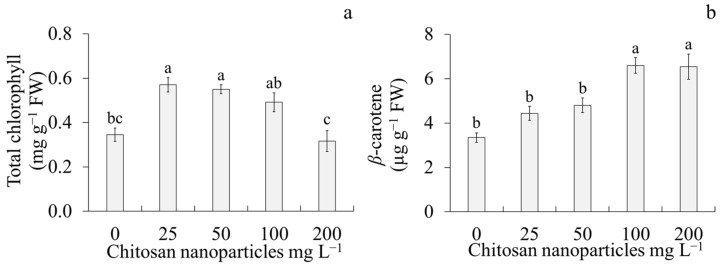
Effect of different chitosan nanoparticle concentrations on photosynthetic pigment contents of sugarcane (*Saccharum* spp.) cv. Mex 69–290. (**a**) Total chlorophyll and (**b**) *β*-carotene after four weeks of culture. Each value represents the mean ± standard error. Means with different letters within a column are significantly different using Tukey’s test (*p* < 0.05). FW: fresh weight.

The different CsNP concentrations had an effect on chlorophyll and *β*-carotene contents (Figure 2). The highest chlorophyll content was observed in the treatments with 25 and 50 mg L^−1^ CsNPs, whereas the lowest chlorophyll content was observed in the treatment with 200 mg L^−1^ CsNPs (Figure 2a). For *β*-carotene, the highest content of this pigment was observed in the treatments with 100 and 200 mg L^−1^ CsNPs, whereas the lowest carotene content was observed in 0, 25, and 50 mg L^−1^ CsNPs (Figure 2b).

### 2.3. Effect of CsNPs on Phenolic Content, Lipid Peroxidation, Hydrogen Peroxide Content, and Antioxidant Capacity

**Figure 3:**
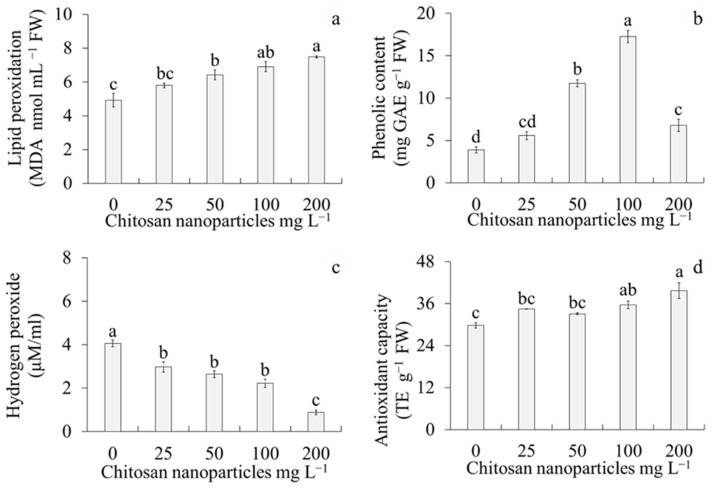
Effect of different chitosan nanoparticle concentrations on in vitro multiplication of sugarcane (*Saccharum* spp.) cv. Mex 69–290. (**a**) Lipid peroxidation expressed in malondialdehyde (MDA) content, (**b**) phenolic contents expressed in gallic acid equivalents (GAE), (**c**) hydrogen peroxide, and (**d**) antioxidant capacity expressed in trolox equivalents (TE) after four weeks of culture. Each value represents the mean ± standard error. Different letters are significantly different according to Tukey’s test (*p* < 0.05). FW: fresh weight.

The different CsNP concentrations influenced hydrogen peroxide content, lipid peroxidation, phenolic content, and antioxidant capacity (Figure 3). For lipid peroxidation, the highest MDA content was observed in the 200 mg L^−1^ CsNP treatment, whereas the lowest MDA content was observed in the control treatment (Figure 3a). For phenolic content, the highest gallic acid equivalents (GAE) content was observed in the 100 mg L^−1^ CsNP treatment, whereas the lowest GAE content was observed in the control treatment (Figure 3b). For hydrogen peroxide content, the highest level was observed in the control treatment, whereas the lowest hydrogen peroxide content was observed with 200 mg L^−1^ CsNPs (Figure 3c). Regarding antioxidant capacity, the highest trolox equivalents (TE) content was observed in the 200 mg L^−1^ CsNP treatment, whereas the lowest TE content was observed in the control treatment (Figure 3d).

### 2.4. Acclimatization

**Figure 4:**
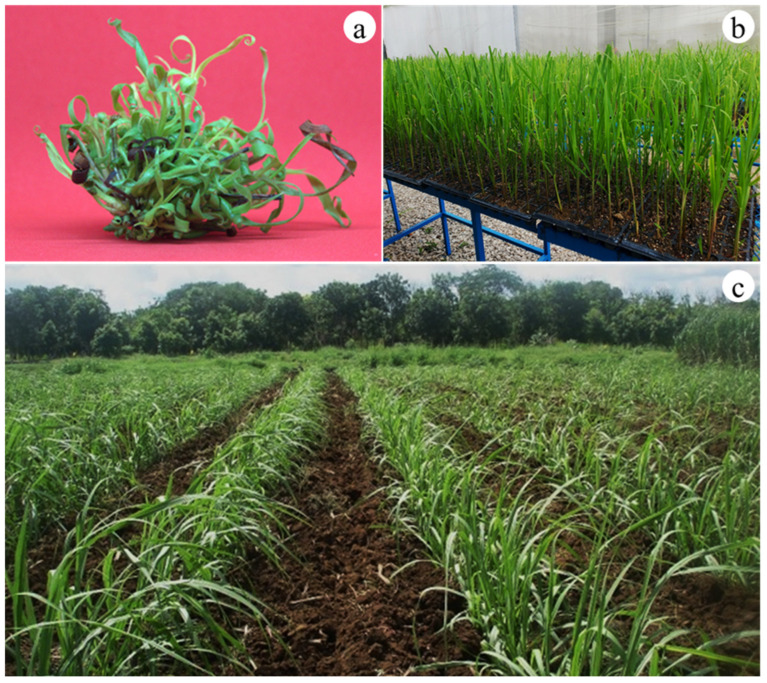
Shoots and plantlets obtained from different chitosan nanoparticle concentrations during ex vitro acclimatization of sugarcane (*Saccharum* spp.) cv. Mex 69–290. (**a**) Ex vitro sugarcane shoots after four weeks of culture, (**b**) sugarcane plantlets in greenhouse after 12 weeks, and (**c**) plantation 15 weeks in field conditions.

At the acclimatization stage, no differences were observed in the survival rates of the different chitosan nanoparticle concentrations evaluated (Figure 4a). *Saccharum* spp. planted showed a survival rate of 99% after 12 weeks of acclimatization (Figure 4b) and were finally transplanted to the field (Figure 4c).

## 3. Discussion

### 3.1. Evaluation of Chitosan Nanoparticles During In Vitro Multiplication

The results obtained in this study showed that CsNPs had an effect on the response percentage, the number of shoots per explant, shoot length, and the number of leaves per explant. The decreased response percentage at doses of 100 and 200 mg L^−1^ CsNPs was probably due to toxicity in the explants at high concentrations of CsNPs. The toxic effect of high concentrations of CsNPs has been reported in the in vitro culture of red pepper (*Capsicum annuum*) [21], potato (*Solanum tuberosum* L.) [16], and gum tragacanth (*Astragalus* spp.) [22]. Asgari-Targhia et al. [21] in *Capsicum annum* reported an inhibition of growth during in vitro seed germination at concentrations of 5, 10, and 20 mg L^−1^ CsNPs. Elsahhar et al. [16] in *Solanum tuberosum* reported a low response percentage during shoot regeneration at concentrations of 300 mg L^−1^ CsNPs. Alhaithloul et al. [22] in *Astragalus* spp. reported a decrease in the percentage of shoot rooting at a concentration of 2 mg L^−1^ CsNPs. The toxicity of CsNPs in *Saccharum* spp. explants can be attributed to an excess in the concentration and/or endogenous accumulation of chitosan in the tissues, causing alterations in homeostasis, generation of ROS, and/or modification of the cellular structure.

On the other hand, in this study, a hormetic effect of CsNPs was observed for the number of shoots per explant, shoot length, and number of leaves per shoot. Hormesis is characterized by the stimulation of development at low concentrations of a stressor and inhibition of development at high concentrations of the stressor [13,23]. The number of shoots per explant increased at doses of 25 and 50 mg L^−1^ CsNPs, whereas the variables shoot length and number of leaves per shoot increased at a dose of 25 mg L^−1^ CsNPs. Inhibition of these developmental variables was observed at doses of 100 and 200 mg L^−1^ CsNPs. The increase in the number of shoots per explant, shoot length, and number of leaves per shoot could be due to the fact that low concentrations of CsNPs can stimulate organogenesis by inducing the synthesis of endogenous phytohormones such as cytokinins and auxins [24], transporting bioactive compounds across cell membranes [18] and improving nutrient uptake from the culture medium [25]. In relation to dry matter, no significant differences were observed between treatments. This fact was probably because the CsNPs (80 ± 10 nm) evaluated in this study are not internalized in tissue cells and have an effect through the apoplast. Alternatively, elements and/or other low molecular weight molecules (hormones, amino acids, metabolites, and/or sugars, among others) could be stored or synthesized, and do not represent a significant difference in weight.

Growth stimulation at low concentrations of CsNPs has been reported in the in vitro culture of potato (*Solanum tuberosum*) [16] and gum tragacanth (*Astragalus* spp.) [22]. Elsahhar et al. [22] reported an increase in the regenerated percentage and shoot length of *Solanum tuberosum* at a concentration of 250 mg L^−1^ CsNPs. Alhaithloul et al. (2024) reported that the rooting percentage of shoots, the number of roots per shoot, and the root length of *Astragalus* spp. increased at a concentration of 0.5 mg L^−1^ CsNPs. The use of CsNPs at concentrations appropriate for each species could stimulate cell growth and differentiation.

### 3.2. Effect of CsNPs on Photosynthetic Pigment

Chlorophyll and carotenoids are important for in vitro photosynthesis. Chlorophyll and carotenoids are pigments present in chloroplasts [26]. *β*-carotenoids play essential roles in photoprotection, pigmentation, and plant signaling, such as regulating gene expression in response to light conditions [27]. Carotenoids have antioxidant properties that neutralize the effects of hydroxyl, peroxyl, singlet oxygen, and superoxide radicals [28,29].

The results obtained in this study showed that CsNPs significantly influence chlorophyll and *β*-carotene content. The increase in chlorophyll content at concentrations of 25 and 50 mg L^−1^ CsNPs could be due to the fact that the application of CsNPs in plants improves photosynthetic activity, induces stomatal functionality through abscisic acid (ABA) synthesis, and enhances antioxidant enzyme activity through nitric oxide (NO) and hydrogen peroxide (H_2_O_2_) signaling pathways [30,31]. Increased chlorophyll content with CsNPs has been reported under ex vitro conditions in corn (*Zea mays*) [32], wheat (*Triticum aestivum*) [33], lupine (*Lupine termis*) [34], garden thyme (*Thymus vulgaris*) [35], and schefflera (*Schefflera arboriecola*) [36]. Khati et al. [32] reported that total chlorophyll content in *Zea mays* seedlings during seed germination increased 1.5–2-fold at 50 mg L^−1^ CsNP concentrations compared to the control. Bakhoum et al. [34] reported increased chlorophyll in *Lupine termis* during seed germination at 50 mg L^−1^ CsNPs. Haghaninia et al. [35] reported increased total chlorophyll in Thymus vulgaris seedlings during their development at 10 mg L^−1^ CsNPs. Imara et al. [36] reported increased total chlorophyll in *Schefflera arboriecola* seedlings at 25 mg L^−1^ CsNPs. In this regard, Chibu and Shibayama [37] note that the nitrogen content in CsNPs can increase the chlorophyll content, since nitrogen is an important element in the tetrapyrrole ring of the chlorophyll molecule.

The increase in *β*-carotene content with CsNPs has been reported under ex vitro conditions in cultures of lupine (*Lupine termis*) [34], garden thyme (*Thymus vulgaris*) [35], and schefflera (*Schefflera arboriecola*) [36]. Bakhoum et al. [34] reported an increase in carotenoid content in *Lupine termis* during seed germination with 50 mg L^−1^ CsNPs. Haghaninia et al. [35] reported an increase in carotenoid content in *Thymus vulgaris* seedlings during their development with 10 mg L^−1^ CsNPs. Imara et al. [36] reported an increase in carotenoid content in *Schefflera arboriecola* seedlings with 25 mg L^−1^ CsNPs.

The application of CsNPs at low concentrations increased chlorophyll content, whereas high concentrations of CsNPs reduced chlorophyll content. This could be explained by the high nitrogen concentrations of CsNPs. Furthermore, chitosan can increase the uptake of minerals such as magnesium and iron, which may contribute to improved chlorophyll synthesis [38]. On the other hand, high concentrations of CsNPs may have caused chlorophyll degradation due to the toxicity of these nanoparticles. Chitosan at low concentrations can restore protein pigment complexes, which help protect the photosynthetic apparatus from oxidative damage [39].

### 3.3. Effect of Chitosan Nanoparticles on Biochemical Parameters

#### 3.3.1. Lipid Peroxidation

Oxidative degradation of lipids, known as lipid peroxidation, is an indicator of oxidative stress [40]. Malondialdehyde (MDA), the byproduct of lipid peroxidation, is widely used as a biochemical marker to assess oxidative stress [41]. In the present study, the increase in MDA content in CsNP treatments may be attributed to possible membrane damage induced by increased CsNP concentrations. Due to their nanometer size, some CsNPs may penetrate cell membranes, increasing the possibility of causing toxicity and oxidative damage. Arif et al. [42] note that high concentrations of CsNPs induce phytotoxic effects, decrease seed germination, and inhibit seedling development. In contrast, Alenazi et al. [43] in bean (*Phaseolus vulgaris*) reported a decrease in lipid peroxidation during seedling development when applying 500 mg L^−1^ of salinity-induced CsNPs. Elevated ROS levels can alter cellular metabolism, leading to increased lipid peroxidation and formation of cytotoxic radicals derived from oxidative stress [44]. This fact could confirm the low response percentages when increasing CsNP concentrations.

#### 3.3.2. Phenolic Content

Phenolic compounds are secondary metabolites with antioxidant properties, cell signaling, and the ability to prevent oxidative stress [45]. Plants activate various biochemical mechanisms in response to oxidative stress, including increased production of flavonoids, phenolic acids, tannins, and hydroxycinnamic acid [46]. The increase in phenolic content at concentrations of 25, 50, and 100 mg L^−1^ CsNPs could be due to the fact that antioxidant signaling and/or defense mechanisms are induced at these CsNP concentrations. According to Hajihashemi et al. [47], phenolic compounds show antioxidant activity by scavenging H_2_O_2_ by donating electrons to guaiacol-type peroxidases under stress conditions. Saini et al. [45] point out that phenolic compounds function as signaling molecules by triggering the activation of stress response genes. On the other hand, the decrease in phenolic content at concentrations of 200 mg L^−1^ CsNPs could be due to a toxic effect caused by an excess of CsNPs, resulting in tissue damage and affecting phenol synthesis. This fact confirms the low response percentage of explants to this concentration. The increase in phenolic content with CsNPs has been reported under ex vitro conditions in wheat (*Triticum aestivum*) [48], lupine (*Lupine termis*) [35], and schefflera (*Schefflera arboriecola*) [36]. Hajihashemi and Kazemi [48] observed an increase in phenol content in *Triticum aestivum* during seed germination with 50 mg L^−1^ CsNPs. Bakhoum et al. [34] reported increased phenol content in *Lupine termis* during seed germination with 50 mg L^−1^ CsNPs. Imara et al. [36] reported an increase in total phenolic content in *Schefflera arboriecola* during seed germination with 10 mg L^−1^ CsNPs. Plant tissues possess phenolic compounds that play vital roles in plant physiology, including development, metabolism, and defense mechanisms. One of the functions of phenolic compounds is their antioxidant capacity. They act as electron donors, neutralizing free radicals generated during oxidative stress [49].

#### 3.3.3. Hydrogen Peroxide

Hydrogen peroxide is an ROS that, at low concentrations, functions as a signaling molecule, participating in physiological processes such as growth and differentiation. However, at high concentrations, it can be toxic to plant cells, causing oxidative stress and cell death. H_2_O_2_ can oxidize lipids, proteins, and nucleic acids [50]. In addition, H_2_O_2_ is involved in the polymerization and formation of lignin in the cell wall, a crucial process for strengthening the cell wall and providing structural integrity [51].

The decrease in H_2_O_2_ content with increasing CsNP concentrations could be attributed to the activation of antioxidant defense mechanisms. Probably, CsNPs induce the activity of enzymatic and non-enzymatic antioxidants to decrease H_2_O_2_ production. According to Ali et al. [52], catalase, peroxidases, peroxiredoxins, thioredoxins, and superoxide dismutase enzymes help to detoxify excess H_2_O_2_ and maintain redox homeostasis. Also, Smirnoff and Arnaud [53] point out that glutathione peroxidase and ascorbate peroxidase counteract H_2_O_2_. Das and Mukherjee [29] note that non-enzymatic antioxidants such as ascorbic acid act in the reduction of H_2_O_2_ during photosynthetic reactions through electron transport. Through the coordinated action of these enzymatic and non-enzymatic antioxidants, plants effectively detoxify excess H_2_O_2_, thereby protecting cellular integrity and function. CsNPs have been shown to reduce oxidative stress in plants by neutralizing H_2_O_2_ and other ROS under ex vitro conditions in mung bean (*Vigna radiata*) [54], periwinkle (*Catharanthus roseus*) [55], and banana (*Musa acuminata*) [56]. Sen et al. [5] reported a decrease in H_2_O_2_ content in *Vigna radiata* during seed germination with 2 mg L^−1^ CsNPs. Hassan et al. [55] reported decreased H_2_O_2_ content in *Catharanthus roseus* during seedling development with 10 mg L^−1^ CsNPs. Wang et al. [56] observed the decrease in H_2_O_2_ content in *Musa acuminata* during seedling development with 100 mg L-1 CsNPs. This study demonstrates that CsNPs can attenuate H_2_O_2_ accumulation under in vitro conditions. In general, ROS accumulation in the absence of antioxidant activity can lead to oxidation of biomolecules, such as protein damage, oxidation of DNA and RNA nucleotides, enzyme inhibition, lipid peroxidation, and activation of apoptosis [51].

#### 3.3.4. Antioxidant Capacity

Antioxidants are molecules capable of inhibiting or eliminating free radical reactions, delaying or preventing cell damage [57]. According to Kiss et al. [58], quantifying the antioxidant capacity of plant tissues is essential for understanding the protective functions of bioactive compounds and neutralizing free radicals. The trolox equivalent (TE) has the ability to determine both the hydrophilic and lipophilic antioxidant capacities of extracts [59]. Trolox protects cells against the toxic effects of H_2_O_2_ by reducing the ability of cells to absorb H_2_O_2_ into their internal structure and the activation of antioxidant enzymes [60]. The increase in antioxidant capacity with increasing CsNP concentrations could be due to the synthesis of enzymatic and non-enzymatic antioxidant compounds (ascorbate, glutathione, phenols, tocopherols, tocotrienols, and fat-soluble carotenoids) that prevent oxidation of biomolecules. In this study, antioxidant capacity is related to the decrease in hydrogen peroxide content. As antioxidant capacity increases, H_2_O_2_ content decreases. The increase in antioxidant capacity content with CsNPs has been reported under ex vitro conditions in bean (*Vicia faba*) [61], lettuce (*Lactuca sativa*) [62], and sea lily (*Pancratium maritimum*) [63]. Dawood et al. [61] reported increased antioxidant capacity in *Vicia faba* during seed germination with 20 mg L^−1^ CsNPs. Ramirez-Rodriguez et al. [62] reported increased antioxidant capacity in *Lactuca sativa* during seed germination with 0.1, 0.2, 0.4, and 0.8 mg L^−1^ CsNPs. Allam et al. [63] reported increased antioxidant capacity in *Pancratium maritimum* during seed germination with 1 mg L^−1^ CsNPs. According to Arif et al. [42], CsNPs mitigate toxicity symptoms under different types of stress by activating antioxidant enzymes.

The results obtained in this study demonstrated that CsNPs have effects on the development, chlorophyll content, *β*-carotene, hydrogen peroxide, lipid peroxidation, phenolic content, and antioxidant capacity of sugarcane during in vitro multiplication. The effects of CsNPs on plants can be attributed to their physicochemical properties, such as size, shape, concentration, color, specific gravity, solubility, and purity. These characteristics influence the response and interactions with plant cells, causing alterations in homeostasis, oxidative stress, ROS generation, and modification of cell structure [64]. CsNPs had a hormetic effect on the number of shoots per explant, shoot length, number of leaves per shoot, and chlorophyll content. It has been shown that the first phase of hormesis is characterized by stimulation at low concentrations of a stressor, whereas the second phase occurs through inhibition at high or lethal concentrations of the stressor. ROS generation is key during hormesis. Excess ROS can trigger programmed cell death, affecting cell growth and differentiation. However, ROS at low concentrations are considered hormetic molecules because they participate in the cell cycle, tolerance to abiotic factors, and genomic integrity, whereas at high concentrations they are toxic to cells and can cause cell death [65]. Phenolic compounds and antioxidant capacity could be associated with the antioxidant response to oxidative stress caused by high concentrations of chitosan nanoparticles. Elevated levels of MDA, together with increased hydrogen peroxide (H_2_O_2_), can inactivate antioxidant enzymes, disrupt redox signaling, and trigger apoptosis [66]. The interaction of MDA and H_2_O_2_ with biomolecules such as lipids, proteins, and DNA leads to structural and functional alterations that severely affect plant development and productivity under stress conditions [67]. Exposing explants to low or sublethal doses of nanochitosan could induce stress tolerance mechanisms in the future.

## 4. Materials and Methods

### 4.1. Plant Material, In Vitro Establishment, and Culture Conditions

Shoot tips 28 cm in length were collected from seven-month-old sugarcane (*Saccharum* spp.) cv. Mex 69–290 plants. The tips were washed with potable water and liquid detergent (Axion^®^ Mission Hills, S.A. de C.V., San José de Iturbide, GTO, Mexico), wrapped in Kraft paper bags (28 × 11 cm), and refrigerated at 4 °C for 24 h. The tips were trimmed to a size of 8–10 cm and subjected to hydrothermotherapy in a circulating thermostatic bath (Ecoshel, SC-15; Pharr, TX, USA)at 50 °C over a period of 8 min. Subsequently, the tips were introduced into a laminar flow hood, cutting them to a length of 2 cm and rinsing them for 7 min in a 0.25% (*v*/*v*) sodium hypochlorite solution with three drops of Tween 20^®^ (Merck KGaA^®^, Darmstadt, DE, USA) per 100 mL of sterile water. The tips were washed four times with sterile water; using them as explants, they were placed in test tubes (22 × 150 mm) containing 15 mL of MS medium [68] supplemented with 30 g L^−1^ sucrose and 1 mg L^−1^ methylene blue (Merck KGaA^®^), without growth regulators. The medium pH was adjusted to 5.8, and 2.3 g L^−1^ Gelrite^®^ (Merck KGaA^®^) was added as a gelling agent and autoclaved at a pressure of 1.5 kg cm^−2^ at 121 °C for 15 min. All explants were incubated at 25 ± 2 °C under a photosynthetic photon flux density of 30 ± 5 μmol m^−2^ s^−1^ and a 16:8 (light: dark) photoperiod.

### 4.2. Evaluation of Chitosan Nanoparticles During In Vitro Shoot Multiplication

Temporary immersion bioreactors (TIBs) with a capacity of 1000 mL were used, and 10 explants of two shoots each were placed in each bioreactor. The TIBs consist of two glass vessels, one of which contains the liquid culture medium and the other the explants. Both vessels are interconnected by a silicone tube, and hydrophobic filters are used to prevent contamination. Explant immersion is achieved using pressure generated by an air compressor, which, with the help of a solenoid valve, propels the liquid medium toward the explants for a programmed time. Furthermore, another solenoid valve reverses the air flow to remove the liquid medium from the explants. A timer regulates the immersion time and frequency by turning the solenoid valves on and off. The TIBs contained 500 mL of liquid MS medium supplemented with 30 g L^−1^ sucrose, 0.6 mg L^−1^ indoleacetic acid (IAA, Merck KGaA^®^),1 mg L^−1^ kinetin (KIN, Merck KGaA^®^), 0.6 mg L^−1^ 6-benzylaminopurine (BAP, PhytoTech Labs, Lenexa, KS, USA), and 1 mg L^−1^ methylene blue (Merck KGaA^®^), according to Sorcia-Morales et al. [69]. Chitosan nanoparticles (NANOCHEMAZONE^®^, Leduc, AB, Canada) were added at different concentrations (0, 25, 50, 100, and 200 mg L^−1^). The CsNPs were obtained by the Synthesis Electrostatic spray method (Electrospraying). The average particle size was about 80 ± 10 nm, and purity was >99.9. Three TIBs were used for each treatment, and the entire experiment was conducted in triplicate (three repetitions × replicated three times = nine repetitions in total). In the TIBs, the immersion frequency was 2 min every 9 h, as proposed by Lorenzo et al. [70]. Finally, the same sterilization and incubation conditions described above were applied.

### 4.3. Chlorophyll (Chl) Determination

Chl content was determined using the protocol described by Spinoso-Castillo et al. [71]. For each treatment, 0.25 g of fresh leaf tissue was macerated in a porcelain mortar using 5 mL of 80% acetone. Each sample was then placed in 10 mL polypropylene tubes (Corning, Inc., Corning, NY, USA) and centrifuged at 3000 rpm for 15 min at 5 °C. The absorbance of 663 and 645 nm for chlorophyll a and b, respectively, was determined using a spectrophotometer (Genesys 10S, Thermo Scientific, Waltham, MA, USA).

Chlorophylls were calculated using the following formulas:

Chl a content (mg g^−1^) = [(12.7 × A_663_) – (2.69 × A_645_)] × [W/(1000 × V)]

Chl b content (mg g^−1^) = [(22.9 ×A_645_) – (4.68 × A_663_)] × W/(1000 × V)]

Total Chl content (mg g^−1^) = Chl a + Chl b

where: A_663_ and A_645_: absorbance, V = volume graduation in mL^−1^, W = sample weight in g, and 1000: conversion factor.

### 4.4. Carotenoid Determination

To determine the carotenoid (*β*-carotene) content, the method described by Biehler et al. [72] was applied. The absorbance at 450 nm was determined using a spectrophotometer (Genesys 10S), and the following formula was used:

C = (A_450_ × M × 1000)/(ε × δ).

where: C indicates the carotenoid content, A_450_ measures the absorption, M represents *β*-carotene (molecular mass, 537 g^−1^ mol), ε refers to the molar extinction coefficient of *β*-carotene in acetone (140.6 L^−1^ mol cm), and δ describes the optical path (cm).

### 4.5. Lipid Peroxidation Determination

For lipid peroxidation (MDA) determination, a colorimetric kit (Chemical^®^, Cambridge, UK) was used. A total of 50 mg of plant material was ground using a mortar and pestle, followed by the addition of 375 µL of sterilized water containing 7.5 µL of butylated hydroxytoluene for each treatment, followed by the addition of 1 vol of 2 N PCA. The sample was shaken and centrifuged at 4000× *g* at 10 °C for 10 min. Then, 100 µL of the supernatant was removed and transferred to a vial containing 100 µL of sodium dodecyl sulfate solution and mixed with 4 mL of color reagent. The samples were incubated for 1 h at 100 °C and centrifuged. Absorbance readings were measured at 532 nm using a UV-Vis spectrophotometer.

### 4.6. Phenolic Content Determination

Phenolic content determination was based on the methodology of Payet et al. [73]. For each sample, 250 mg of fresh plant tissue was weighed and macerated in a mortar using 5 mL of methanol and water 80:20 (*v*/*v*). The mixture was centrifuged at 4000× *g* at 10 °C for 10 min. A total of 150 µL of the supernatant was removed by adding 750 µL of 10% Folin–Ciocalteual reagent (Merck KGaA^®^) and 600 µL of 20% sodium carbonate (Merck KGaA^®^). The samples were then left to stand for 4 h in the dark at 27 °C. Absorbance was recorded at 765 nm and read with a UV-Vis spectrophotometer (Genesys 10S Thermo Fischer Scientific, Waltham, MA, USA).

### 4.7. Hydrogen Peroxide Content

A colorimetric and fluorometric assay was used to measure hydrogen peroxide (H_2_O_2_) as a reactive metabolic byproduct of oxygen (O_2_). The Peroxide Assay kit (CRT scientific, Monterrey, N.L., Mexico) was used. The determination was performed as indicated by the supplier. First, 50 mg of plant material was deproteinized for each sample, macerated with perchloric acid (PCA), and then 800 µL of potassium hydroxide was added to neutralize the sample. The mixture was centrifuged at 4000× *g* at 10 °C for 10 min. Next, 1 mL of the supernatant was collected to add 3400 µL of reagent A, and then 34 µL of reagent B was added while stirring. Subsequently, 40 µL of the supernatant was collected, and 200 µL of reagents A and B were added, and the sample was allowed to stand for 30 min. All samples were run in triplicate. Absorbances were read at 585 nm and read with a UV-Vis spectrophotometer (Synergy HT, BioTek, Winooski, VM, USA). 

### 4.8. Antioxidant Capacity

Determination Antioxidant capacity was determined according to the protocol proposed by Huang et al. [74]. For each treatment, 100 µL of the methanolic extract obtained from the soluble phenol analysis was added. Peroxide radicals were generated by DPPH (2,2-Diphenyl-1-picrylhydrazyl), and absorbance was recorded at 515 nm.

### 4.9. Experimental Design and Statistical Analysis

All experiments were laid out in a completely randomized design with three replicates. After four weeks of culture, the number of shoots per explant, shoot length, number of leaves per explant, dry matter, chlorophyll content, *β*-carotene content, phenolic content, antioxidant capacity, lipid peroxidation, and hydrogen peroxide content were evaluated. The Kolmogorov–Smirnov and Levene’s tests were used to determine whether the data had a normal distribution and homogeneity of variance. Data were analyzed with ANOVA (analysis of variance) and Tukey’s range test (*p* < 0.05) using SPSS (Statistical Package for the Social Sciences, v23) statistical software. An analysis of variance was performed with the transformed values using the formula Y = arcsine (√ (x/100)), where “x” is the percentage value.

## 5. Conclusions

In conclusion, chitosan nanoparticles can induce a hormetic effect during in vitro multiplication of sugarcane. Studies are suggested to elucidate other biochemical and molecular mechanisms in response to the use of chitosan nanoparticles for other potential applications in plant tissue culture.

## Figures and Tables

**Table 1:** Effect of different chitosan nanoparticle concentrations on in vitro multiplication of sugarcane (*Saccharum* spp.) cv. Mex 69–290 after four weeks of culture.

Chitosan Nanoparticles (mg L^−1^)	Response (%)	Number of Shoots Per Explant	Shoot Length (cm)	Number of Leaves Per Shoot	Fresh Weight (g)	Dry Weight (g)	Dry Matter (%)
0	100 ± 0.00 a	26.33 ± 0.88 b	3.81 ± 0.12 c	3.48 ± 0.13 bc	2.68 ± 0.31 b	0.23 ± 0.02 ab	8.93 ± 0.29 a
25	100 ± 0.00 a	32.42 ± 1.10 a	5.78 ± 0.09 a	4.38 ± 0.12 a	5.53 ± 1.03 a	0.48 ± 0.07 a	9.00 ± 0.53 a
50	100 ± 0.00 a	34.33 ± 1.40 a	5.00 ± 0.15 b	3.68 ± 0.14 b	3.71 ± 0.61 ab	0.44 ± 0.07 ab	11.56 ± 2.42 a
100	83 ± 3.33 b	18.00 ± 1.96 c	2.56 ± 0.11 d	3.00 ± 0.10 c	2.52 ±0.32 b	0.26 ± 0.10 ab	9.60 ± 2.56 a
200	66 ± 3.33 c	9.42 ± 0.36 d	2.10 ± 0.09 d	2.37 ± 0.11 d	1.79 ± 0.09 b	0.16 ± 0.01 b	9.02 ± 0.92 a

Each value represents the mean ± standard error. Different letters indicate significant differences (Tukey, *p* < 0.05).

## Data Availability

The data can be made available upon request.
